# Comparing synthetic mammograms based on wide-angle digital breast tomosynthesis with digital mammograms

**DOI:** 10.1117/1.JMI.12.S1.S13011

**Published:** 2025-01-20

**Authors:** Magnus Dustler, Gustav Hellgren, Pontus Timberg

**Affiliations:** a Lund University, Department of Translational Medicine, Diagnostic Radiology, Malmö, Sweden; b Lund University, Department of Translational Medicine, Medical Radiation Physics, Malmö, Sweden; c Skåne University Hospital, Radiation Physics, Malmö, Sweden

**Keywords:** synthetic mammography, digital breast tomosynthesis, visual grading, prior mammography, visual grading characteristic, digital mammography

## Abstract

**Purpose:**

We aim to investigate the characteristics and evaluate the performance of synthetic mammograms (SMs) based on wide-angle digital breast tomosynthesis (DBT) compared with digital mammography (DM).

**Approach:**

Fifty cases with both synthetic and digital mammograms were selected from the Malmö Breast Tomosynthesis Screening Trial. They were categorized into five groups consisting of normal cases and recalled cases with false-positive and true-positive findings from DM and DBT only. The DBT system used was a wide-angle (WA) system from Siemens, and the SM images were reconstructed from the DBT images. Visual grading, detection, and recall were evaluated by experienced breast radiologists in both SM and DM images.

**Results:**

Some image quality criteria of the SM images were rated as qualitatively inferior to DM images. However, reader-averaged diagnostic accuracy (0.57 versus 0.55), sensitivity (0.46 versus 0.50), and specificity (0.64 versus 0.58) were not significantly different between SM and DM, respectively

**Conclusions:**

Synthetic mammography plays a promising role to complement or even replace DM. The study could not find any indications of substantial differences in the sensitivity or specificity of SM for WA DBT systems compared with DM. However, certain image quality criteria of SM fall slightly short compared with DM images. Next-generation DBT systems could address such limitations through improved reconstruction algorithms and system design, and their performance should be the focus of future research studies.

## 1 Introduction

A widely accepted method in breast cancer screening is full-field digital mammography (FFDM) where craniocaudal (CC) and mediolateral oblique (MLO) views are collected.^1^ However, two-dimensional imaging of the breast could potentially result in tissue overlap that may hide malignant findings or falsely appear as such. To reduce this effect, digital breast tomosynthesis (DBT) has been proposed. It is a pseudo-three-dimensional imaging technique where the X-ray tube moves in a limited angular span while acquiring projection images of the breast to be used to reconstruct slices.^2^ Recent data have shown that DBT when implemented in a screening program increases breast cancer detection rates.^3^^–^^6^

When DBT was first introduced, to speed up regulatory approval, it was evaluated in a setting combined with digital mammography (DM) to not show inferior performance. However, this resulted in additional radiation dose, and therefore, a technique to generate two-dimensional (2D) synthetic mammograms (SMs) based on already acquired DBT image data was developed. The appearance of an SM closely resembles a regular DM, and a recent review showed not only the implementation and benefits but also some drawbacks.^7^

The angular span used in DBT determines the effective in-depth resolution of the DBT volume, with wide-angle (WA) systems resulting in a higher in-depth resolution compared with narrow-angle (NA) systems. Most large clinical studies that evaluated DBT in screening did this as an adjunct to DM (or SM), and not as a standalone modality, and used NA-DBT and not WA-DBT systems.^8^^,^^9^ One exception is the Malmö Breast Tomosynthesis Screening Trial (MBTST) where one-view WA-based DBT (in the MLO projection) was compared with two-view FFDM (MLO + CC).^10^ It was shown that 34% more cancers were detected using DBT alone compared with DM—indicating that DBT volumes in MLO may be sufficient to increase cancer detection without additional scan time and patient dose from extra DMs or the CC projection with DBT. DBT as a standalone modality resulted however in increased false-positive (FP) recall rate compared with DM. This could be due to uncertainties introduced when lacking a two-dimensional overview of the breast, along with not being able to make direct comparisons with prior screening mammograms.

There are several studies where SM is compared with DM alongside DBT with equivalent results;^11^^,^^12^ however, most of them are focused on SM based on NA-DBT systems. The MBTST dataset provides a unique opportunity to conduct such studies on WA-DBT; the purpose of this study was to investigate the characteristics, detection, and recall rate using SM based on WA-DBT and to compare it with DM. This study investigates the differences in image quality between SM and DM images using recently validated image quality criteria.^13^ Secondary aims were to determine future avenues of improvement for the SM reconstruction algorithm and to investigate the relative changes in specificity and sensitivity compared with standard DM.

## 2 Material and Methods

This study involves the evaluation of detection, recall, and visual grading of SM images and comparisons with DM. The images used in this study were selected from the prospective, population-based MBTST where 14,848 women were screened with both two-view DM and one-view DBT (NCT01091545).^14^ These images had previously been acquired with a wide-angle MAMMOMAT Inspiration DBT system, and the SM images were retrospectively reconstructed using Insight 2D (Siemens Healthineers, Forchheim, Germany). The study was approved by the Local Ethics Committee at Lund University (Official Records No. 2009/770).

To investigate the characteristics of the SM images, four breast radiologists (>7 years of experience reading screening and clinical mammograms) read the same 50 selected cases from the MBTST. The study was based on a comparison between SM and standard DM from the same screening occasion. The image material was stratified into five distinct groups according to the original screening results, with each group of images selected as a random sample of eligible cases from the MBTST dataset. There were 10 cases each of non-recalled normal, false positives recalled on only DBT, false positives recalled only on DM, cancers recalled only on DBT, and cancers recalled only on DM. To not disadvantage readers in the study and to obtain comparable reading results, it was decided to use the screening results of the first screen reader only, instead of the final double reading decision.

The study was set up as two different reading tasks, with the same set of images. All cases were read on a Barco clinical workstation monitor (Barco NV, Kortrijk, Belgium) running ViewDex 2.0 software—a viewing software that has been developed for setting up observer performance studies.^15^^,^^16^ The readers were allowed to use built-in tools for panning, zooming, and window leveling. To analyze the malignancy data, a multireader multicase receiver operating characteristics (MRMC ROC) analysis of covariance toolbox (MRMCaov)^17^ for MATLAB (MathWorks, MATLAB version R2023b) was used to calculate the area under the curve (AUC) and to test for the statistical differences among the modalities. MRMC ROC handles multiple readers and cases and uses the jackknife resampling technique to calculate confidence intervals. In this case, the readers and cases were treated as random factors.

### 2.1 Detection and Recall

For the detection study, each of the readers read right and left MLO views of the same case, SM in one session, and DM in one session. Cases were presented in random order, so no two readers had the same order of cases and also had a different case order in their individual SM and DM sessions. There was at least a 2-week washout period among sessions. The readers’ task was to decide if the case should be recalled or not and to rate its risk of malignancy on a continuous confidence scale from 1 to 10 (1: no risk and 10: highest risk). Sensitivity and specificity were calculated based on recall decision and ROC curves were constructed using the risk of malignancy score. As a threshold for a considered recall among the readers, the case was considered recalled if at least two readers marked recall.

### 2.2 Image Quality

To assess the image quality differences between SM and DM images, a set of 10 relevant criteria were selected from the 18 validated qualitative image quality criteria developed by Boita et al.^13^^,^^18^ These criteria were derived to reflect the image quality relevant for the clinical performance task rather than falling into a trap of a “beauty contest.” In this study, criteria related to breast positioning were excluded as they are not relevant to the task. After consulting directly with Boita et al., the detailed criteria for different aspects of calcification and soft tissue lesion characteristics were simplified and combined to focus on lesion and microcalcification visibility and margins.

The following final set of 10 criteria were used:

1. The texture of glandular and adipose tissues is depicted appropriately in the image.
2. The grayscale depiction of glandular tissues is appropriate.
3. The distinction between adipose and glandular tissues is appropriate.
4. Normal structures such as the Cooper’s ligaments, ducts, and vessels are depicted sharply.
5. The grayscale depiction of the pectoralis muscle is appropriate.
6. The grayscale depiction of the skin line is appropriate.
7. Calcifications are visible.
8. Calcifications are distinguishable from each other.
9. Soft tissue lesions are visible.
10. The margins (including spiculations) of soft tissue lesions are visible.

Note: Criterion 7 refers mainly to the visibility of microcalcification clusters, not individual microcalcifications.

All readers read SM and DM images of the same case side-by-side and were asked to rate the relative quality of images according to the 10 criteria. A relative scale from −2 to 2 was used (−2, left image superior; −1, left image somewhat better; 0, images equal; 1, right image somewhat better; and 2, right image superior). The order of images (SM or DM to the left or right of the screen) was randomized.

The results were analyzed using visual grading characteristic (VGC) statistics^19^ employing VGC Analyzer 1.0.2.^20^ VGC is a statistical tool made for non-parametric, rank-invariant tests that analyses the input data similarly to an ROC study. It is designed for visual grading on an absolute scale but has previously been used by the developers to analyze similar relative ranking data to the one used in this study.^21^^,^^22^ It produces a unit curve relating a test condition to a reference condition. An AUC >0.5 means that the test condition is rated higher, and an AUC <0.5 means that the reference condition is rated higher. For the study, DM was set as the reference condition and SM as the test condition. The software uses bootstrapping to create confidence intervals for the AUC output. A fixed-reader analysis was made for each calculation, along with a binormal AUC curve fitting. The presented p-values in this work are based on a fixed-reader analysis using a binormal curve fit. The method also employs bootstrapping statistical resampling to get confidence intervals of the AUC using 2000 iterations. IBM SPSS Statistics 25.0.0.2 was used for all other statistical calculations. As the VGC statistic requires that all readers rate all cases, if any criterion was rated as “not applicable” by at least one reader for any one case, that case was excluded from the analysis of that specific criteria. This meant practically that cases in which, for example, no clusters of microcalcifications were present in neither the DM nor SM image were not rated for criteria 7 and 8.

## 3 Results

### 3.1 Detection and Recall

**Fig. 1:**
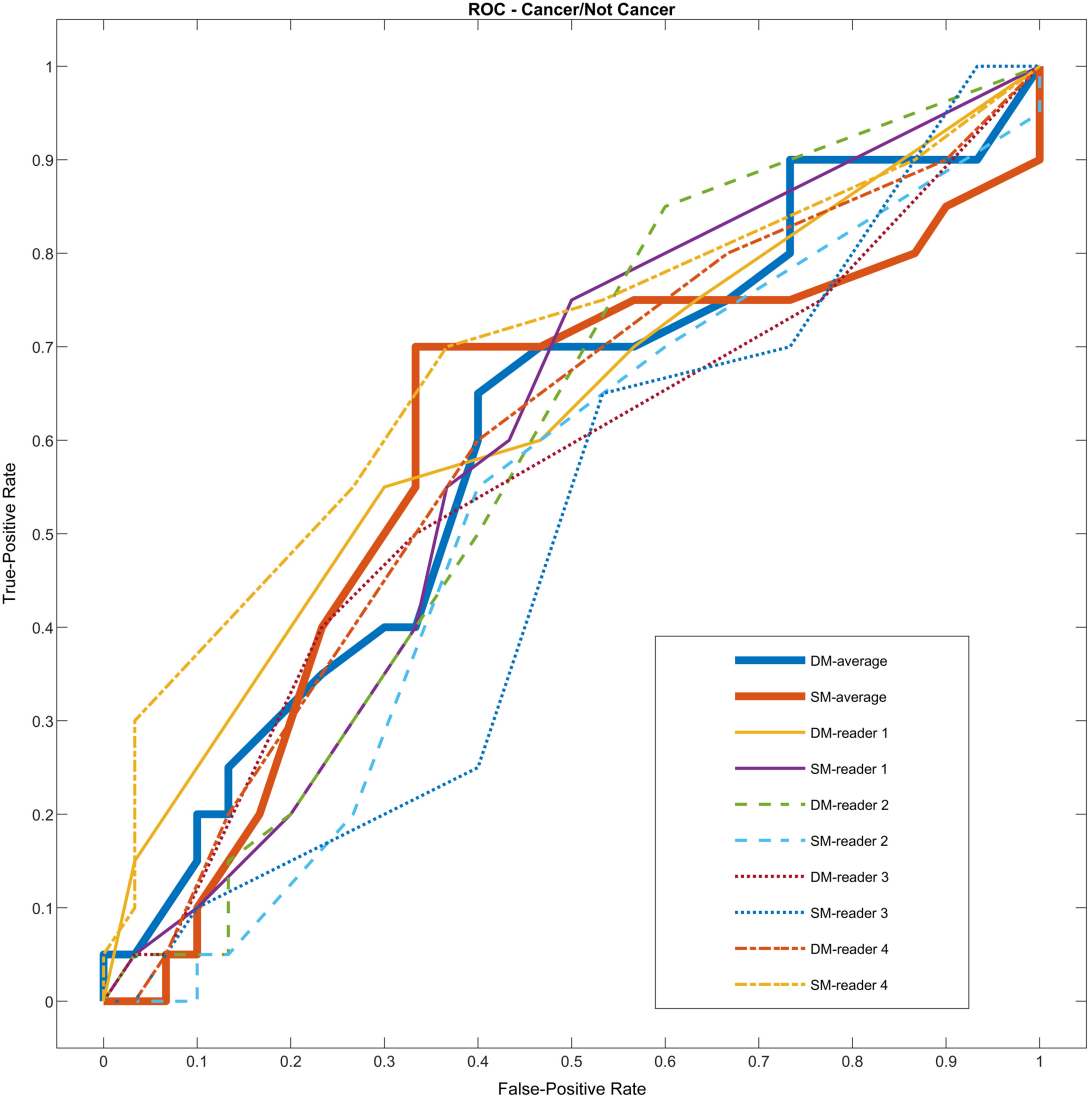
ROC curves of the SM and DM sets for each reader and their average.

To begin, we summarized the outcomes related to detection performance and recalls for each reader and their average in Tables 1 and 2. ROC curves are also shown in Fig. 1 showing the detection performance for these groups (the AUC of each curve is presented in the tables). Considering the mean pairwise difference in AUC among the modalities, a p-value of 0.74 was achieved.

**Table 1:** Individual and average reader results for DM showing recalls per category, sensitivity, specificity, and AUC. Ninety-five percent binomial confidence intervals for both sensitivity and specificity were calculated based on the Clopper–Pearson method. For the individual categories (columns 4 to 8), the fraction of correctly identified cases is shown.

	Sens.	Spec.	Non-recalled normal^a^	FP DBT only^a^	FP DM only^a^	Cancer DBT only^a^	Cancers DM only^a^	AUC
Reader 1	55% (32 to 77)	57% (37 to 75)	10/10	5/10	2/10	6/10	5/10	0.60
Reader 2	30% (12 to 54)	70% (51 to 85)	10/10	7/10	4/10	3/10	3/10	0.56
Reader 3	65% (41 to 85)	43% (25 to 63)	6/10	5/10	2/10	7/10	6/10	0.63
Reader 4	50% (27 to 73)	60% (41 to 77)	9/10	4/10	5/10	6/10	4/10	0.60
DM: pooled reader^b^	50% (39 to 61)	58% (28 to 66)	35/40	21/40	13/40	22/40	18/40	0.60 (0.48 to 0.70)

a Correctly identified cases.

b Pooled results of all readers, representing an average reader.

**Table 2:** Individual and average reader results for SM showing recalls per category, sensitivity, specificity, and AUC. Ninety-five percent binomial confidence intervals for both sensitivity and specificity were calculated based on the Clopper–Pearson method. For the individual categories (columns 4 to 8), the fraction of correctly identified cases is shown.

	Sens.	Spec.	Non-recalled normal^a^	FP DBT only^a^	FP DM only^a^	Cancer DBT only^a^	Cancers DM only^a^	AUC
Reader 1	45% (23 to 68)	73% (54 to 88)	8/10	9/10	5/10	4/10	5/10	0.69
Reader 2	25% (9 to 49)	63% (44 to 80)	8/10	7/10	4/10	3/10	2/10	0.49
Reader 3	60% (36 to 81)	63% (44 to 80)	10/10	6/10	3/10	8/10	4/10	0.60
Reader 4	55% (32 to 77)	57% (37 to 75)	7/10	6/10	4/10	6/10	5/10	0.52
SM: pooled reader^b^	46% (35 to 58)	64% (55 to 73)	33/40	28/40	16/40	21/40	16/40	0.59 (0.42 to 0.73)

a Correctly identified cases

b Pooled results of all readers, representing an average reader

### 3.2 Image Quality

In this part, results from the VGC analysis are shown in Table 3 for each image quality criterion. There were minor statistically significant differences showing that SM was considered less appropriate for the distinction between adipose and glandular tissue and normal structures were not as sharply depicted.

**Table 3:** VGC results for each image quality criterion including 95% confidence intervals and p-values.

	Area under VGC curve	p-value
1. Gland adipose (texture)	0.47 (0.41 to 0.53)	0.63
2. Gland adipose (grayscale)	0.49 (0.44 to 0.55)	0.83
3. Gland adipose (distinction)	0.39 (0.34 to 0.45)	0.01
4. Ligament duct vessels (sharpness)	0.42 (0.36 to 0.49)	0.045
5. Pectoralis (grayscale)	0.47 (0.37 to 0.56)	0.51
6. Skin line (grayscale)	0.48 (0.42 to 0.54)	0.49
7. Calc (visibility)	0.49 (0.42 to 0.55)	0.56
8. Calc (distinguishable)	0.49 (0.38 to 0.62)	0.92
9. Soft tissue lesions (visibility)	0.48 (0.37 to 0.59)	0.84
10. Soft tissue lesion (margins)	0.49 (0.32 to 0.66)	0.89

## 4 Discussion

From the VGC analysis, it is apparent that the two image sets have very similar characteristics. Only two out of 10 questions showed a significant difference, where SM is rated worse on the criteria “the distinction between adipose and glandular tissues is appropriate” and “normal structures such as the Cooper’s ligaments, ducts, and vessels are depicted sharply.” Differences are minor, but this indicates that there is room for improvement in these categories. As there is no difference in the grayscale depiction of glandular tissue, one can speculate that this effect is caused by low sharpness in fine strands, whether fibrous or otherwise. Next-generation DBT systems could address such limitations through improved reconstruction algorithms, and their performance should be the focus of future research studies.^23^

It should be noted that the quality criteria have been validated specifically for DM images, which could potentially lead to bias in their interpretation when applied to SM images. It is unknown how they directly translate to screening performance, although they were developed specifically to cover the image features that are considered by radiologists to be most relevant in the clinical review of mammograms. Considering that SM, despite its differences from a standard DM image, remains a 2D X-ray image of a breast and that it is still primarily intended to visualize changes in breast tissue connected to the presence of breast cancer, in the absence of data to the contrary we have in this study assumed that the image quality criteria relevant to its interpretation can be considered to be the same as for standard DM. This is a limitation of the study, and it could be that separate, tailored image quality criteria need to be adopted for SM images.

In contrast to the literature, there was no indication of any differences in the quality of depiction of microcalcifications, which has been a main reason for combining DBT with DM in the first place, as DBT and SM is believed to be somewhat inferior in calc visibility, as has been shown in, for example, comparative studies on anthropomorphic phantoms.^24^^,^^25^ The current study included a randomized sample of clinical cases. In 26 cases, readers agreed that there were visible calcs that could be rated, which does not necessarily mean that these calcs were related to a suspicious area. A targeted study on cases recalled because of microcalcifications would be valuable to further investigate this.

In contrast to the VGC results, the average specificity was slightly higher for SM than DM, whereas sensitivity was slightly higher for DM, and AUC was the same (0.59 compared with 0.60). None of the differences was statistically significant. The performance difference showed an inter-reader variability, with two of four readers improving specificity and one of four improving sensitivity on SM. Split into different groups of cases, SM notably recalled fewer of the cases that had been false positives on DM but not DBT while also recalling fewer cases that had been false positives on DBT but not DM. SM detected less cancers, both in the DBT-only and DM-only groups. It must be stressed that these results are based on very small groups, but the FP results might indicate that SM manages to combine some of the beneficial aspects of DBT and DM in to resolve cases that would otherwise have been recalled. This warrants further investigation.

A study by Khanani et al.^26^ compared the performance of DBT plus SM against DM using the same WA DBT system as in this study. They showed superior performance in terms of sensitivity and reduced recall. It is however not recommended to compare our results of sensitivity and specificity with other studies as the image set was highly enriched, especially in DBT-only detected cancers. Also, considering the substantial differences in both acquisition geometry and reconstruction methods among different vendors, it is not necessarily possible for the results of either Khanani et al. or of the current study to be generalized to SM from either other wide-angle DBT systems or from DBT systems in general.

As mentioned previously, the aim of this study was to detect substantial differences between DM and SM apparent even with relatively few cases and readers. The image set was selected to include a mix of cases where differences in image quality and appearance can be thought to be especially important, such as false positives. Though not conclusive, the fact that individually there were readers whose relative sensitivity and specificity both increased and decreased for SM reading compared with DM reading suggests that the difference in clinical image quality is quite minor.

A limitation of the study is the analysis of relative image quality rating data using VGC analysis, which normally requires the use of an absolute rating scale, i.e., that each image is rated separately and not relative to another image, to be considered ordinal. This will affect the statistical reliability of the method, but the actual effect is mitigated by the use of clinically accepted DM images acquired using the same automatic exposure control settings as a reference. These images are treated as a constant point of reference for image quality to which the image quality of the SM images is compared using a relative rating. The data are therefore seen as essentially ordinal. A similar approach has been used previously by the designers of VGC statistics.^21^^,^^22^

An important question is the intended use of SM and the implementation of it in a screening program. This study supports that it can be used interchangeably with DM and could be used as a quick overview (maybe with AI CAD overlays) and comparison with priors but also that structures depicted in the DBT image volumes still hold more diagnostic information and should not be replaced by SM. SM images should not be read before DBT to speed up reading by identifying difficult areas, or alone, as the 2D modalities are still clearly inferior. This would lead to missed cancers by not using the full potential of DBT.

## 5 Conclusion

The study could not find any indications of substantial differences in the sensitivity or specificity of SM for WA DBT systems compared with DM. However certain image quality criteria of SM fall slightly short compared with DM images. Next-generation DBT systems could address such limitations through improved reconstruction algorithms and system design, and their performance should be the focus of future research studies.

## Data Availability

Data may be requested by email to the corresponding author. Any access to or transfers of data must however fulfill the necessary requirements such as local regulations, ethics, and law.
